# Obesity Increases Prevalence of Colonic Adenomas at Screening Colonoscopy: A Canadian Community-Based Study

**DOI:** 10.1155/2017/8750967

**Published:** 2017-07-11

**Authors:** Theodore F. Shapero, Grant I. Chen, Tim Devlin, Alison Gibbs, Iain C. Murray, Stanley Tran, Corey Weigensberg

**Affiliations:** ^1^Department of Medicine, The Scarborough Hospital, Scarborough, ON, Canada; ^2^Department of Statistical Sciences, University of Toronto, Toronto, ON, Canada; ^3^Intestinal Health Institute, Markham, ON, Canada; ^4^Department of Medicine, The Scarborough Hospital and North Toronto Endoscopy Clinic, Scarborough, ON, Canada; ^5^Department of Pathology, The Scarborough Hospital, Scarborough, ON, Canada

## Abstract

**Background and Aims:**

Obesity is a risk factor for colorectal neoplasia. We examined the influence of obesity and metabolic syndrome (MetS) on prevalence of neoplasia at screening colonoscopy.

**Methods:**

We evaluated 2020 subjects undergoing first screening colonoscopy. Body mass index (BMI) was calculated at enrolment. Hyperlipidemia (HL), hypertension (HT), and diabetes mellitus (DM) were identified. Details of colonoscopy, polypectomy, and histology were recorded. Odds for adenomas (A) and advanced adenomas (ADV) in overweight (BMI 25.1–30) and obese (BMI > 30) subjects were assessed by multinomial regression, adjusted for covariates. Analyses included relationships between HL, HT, DM, age, tobacco usage, and neoplasia. Discriminatory power of HT, HL, DM, and BMI for neoplasia was assessed by binary logistic regression. Odds were calculated for neoplasia in each colonic segment related to BMI.

**Results:**

A and ADV were commoner in overweight and obese males, obese females, older subjects, and smokers. HL, HT, and DM were associated with increased odds for neoplasia, significantly for A with hypertension. BMI alone predicted neoplasia as well as HT, HL, DM, or combinations thereof. All segments of the colon were affected. Multiple polyps were particularly prevalent in the obese.

**Conclusions:**

Obesity and MetS are risk factors for colonic neoplasia in a Canadian population.

## 1. Background

Colorectal cancer (CRC) is the third most common cancer in Canada and the second and third commonest fatal cancer in men and women, respectively [1]. Several modifiable factors have been suspected to increase susceptibility to CRC. Of these, increased weight and smoking have been strongly implicated [2, 3].

Obesity is increasingly prevalent in Canadians. In 2014, 62% of males and 46% of females were self-reportedly either overweight (BMI 25 to 30 kg/m^2^) or obese (BMI > 30 mg/kg^2^) [4]. If CRC risk indeed increases with weight, the burden of CRC in Canada will be substantially augmented as the population becomes heavier. In that case, lifestyle counselling might ameliorate the risk or the overweight population might be targeted for enhanced CRC screening.

CRC evolves via the adenoma-carcinoma sequence. Modifiable factors might influence CRC incidence through an increase in adenoma formation, an increased conversion rate of adenoma to carcinoma, or an amplification of alternate pathways. Studies carried out elsewhere describe an increased prevalence of both nonadvanced and advanced adenomas with increasing BMI [5, 6], suggesting that obesity promotes neoplastic change at an early stage by increasing adenoma formation.

The mechanisms whereby obesity might promote colonic carcinogenesis are complex, involving insulin resistance, hyperinsulinemia, insulin-like growth factor, adipokines, and inflammation [7]. Insulin resistance and hyperinsulinemia are the foundation of the metabolic syndrome (MetS), whose elements include obesity, type II diabetes mellitus (DM), hyperlipidemia (HL), and hypertension (HT). Earlier studies indicate an increased risk for CRC in persons with MetS [8] as well as an incrementally increased risk for CRC and colorectal adenomas with the number of elements of MetS present [9, 10] suggesting that MetS might be included in the list of CRC risk factors. However, others have not found such an association [11].

Risk stratification for CRC assigns subjects to average versus high risk categories. There is controversy about the most cost-effective screening strategy for average-risk individuals. Controlled trials demonstrating the superiority of colonoscopy are in progress. In the meantime, some jurisdictions, including our own, advocate for fecal occult blood testing or flexible sigmoidoscopy as proven entities [12]. Even so, it is agreed that subjects considered at high risk for CRC would be better served by colonoscopy [13]. This would currently apply to those with a significant family history of CRC or a personal history of colonic neoplasia. Other risk factors, including male sex, ethnicity, tobacco usage, and obesity are not considered sufficiently influential to justify primary colonoscopy. Should any of these factors prove to be strongly associated with CRC, affected individuals might be preferentially assigned to colonoscopy.

This study was designed to examine the relationship between BMI and colonic neoplasia in a Canadian population undergoing screening colonoscopy. The secondary objective was to determine which components of MetS are associated with adenoma prevalence.

## 2. Methods

This prospective, community-based, multicenter study was carried out at The Scarborough Hospital, General and Birchmount Sites, Scarborough, Ontario, The North Toronto Endoscopy Clinic, and The Intestinal Health Institute, Markham, Ontario. Scarborough and Markham are contiguous, ethnically diverse suburbs of Toronto. Subjects were recruited between 2009 and 2014 from ambulatory endoscopy clinics of five participating gastroenterologists. All persons referred to the participating clinics for colonoscopy were surveyed for eligibility. Eligible persons were asymptomatic, aged 40 or older, and undergoing their first colonoscopy. Exclusion criteria included a history of colonic neoplasia, any prior colonoscopy and occult blood in stool. Informed consent was obtained. Participation rate exceeded 95%.

The following data were collected at the time of enrolment: age, sex, family history of CRC in a first degree relative 60 years or younger, and a list of medications. DM, HL, or HT were identified from comorbidities listed in notes from referring physician and direct questioning of subjects in conjunction with a review of medications. The diagnoses were established solely on the basis of historical information as indicated, without refinement by blood testing or other potential confirmatory measures. Smoking was defined as currently consuming any amount of tobacco. Prior smokers were included as nonsmokers. Weight and height were measured at the time of enrolment. BMI was calculated as kg/m^2^. Normal weight was defined as BMI < 25.1, overweight 25.1 to 30, and obesity > 30.

All endoscopists were community gastroenterologists with more than 10 years' experience and an annual volume of colonoscopies exceeding 500. Bowel preparation and colonoscopy were carried out in the customary manner of the participating endoscopist. Data was recorded on whether the preparation was considered satisfactory or inadequate, completeness of procedure and size and location of any lesions excised and retrieved. For polyp location, the colon was divided into 3 segments: rectum, left colon (distal to splenic flexure), and right colon. Polyps were classified as adenoma (A) or advanced adenoma (ADV). Hyperplastic polyps were considered nonneoplastic. ADV was defined as any adenoma 10 mm or larger, or with greater than 25% villous component or high-grade dysplasia or cancer. Serrated adenomas were considered A if less than 10 mm and ADV if 10 mm or larger in size. Subjects were categorized by the most advanced lesion identified. The histologic diagnosis of the reporting pathologist was accepted as accurate. However, a sample of specimens reported at the Scarborough Hospital was reviewed by the reference pathologist (CW) for quality assurance. There was concordance with the reporting pathologist on virtually all cases.

The study was approved by the Research and Ethics Board of The Scarborough Hospital

### 2.1. Statistical Analyses

All analyses were carried out in R version 3.2.2. Multinomial logistic regression models were used to estimate odds ratios (OR), including their confidence intervals and statistical significance from Wald tests. OR were of A or ADV relative to no adenoma. In some analyses, due to insufficient observed numbers of ADV, A and ADV were combined into one category and binary logistic regression was carried out.

Primary analysis examined the effect of BMI. All secondary analyses included adjustment for covariates that were found to be significantly associated with odds of neoplasia. Interactions of these covariates with BMI were examined and not reported due to lack of significance. Each potential covariate was examined individually for its relationship with the odds of A or ADV, and secondary analyses included adjustment for covariates that were found to be significantly associated with odds of neoplasia (*p* < 0.05).

For consideration of polyp location, a model with location as outcome included only participants with at most one affected segment, and a model with the number of segments in which a neoplasm was discovered as the outcome included all participants with complete data.

Discriminatory power of DM, HL, HT, and BMI on the odds of any adenoma was evaluated using area under the receiver operating characteristic curve for the binary logistic regression model. Areas under the curves (AUC) were compared using the DeLong test.

## 3. Results

The study group included 2020 persons. Four subjects missing anthropometrics were excluded from analysis, leaving 2016 subjects with complete data. Colonoscopy was completed to the caecum in 99% of examinations. Prep was considered satisfactory in 97%. The study group is described in Table 1. For males, 71.5% were either overweight or obese, compared to 56.6% of females. One in four of either sex was obese. HT, HL, DM, combinations thereof, and tobacco usage were common and more prevalent in males.

Colonoscopy was normal in 1563 persons. A was detected in 383 persons (18.8%) and ADV in 99 (4.9%), for an overall adenoma detection rate of 23.7%. In the normal colonoscopy group, 37% were of normal weight, 38% were overweight, and 24% were obese, compared with 28%, 40%, and 32%, respectively, for persons with A, and 25%, 47%, and 28% for persons with ADV (Table 2). The risk for A increased progressively with BMI category. In overweight persons, the OR for A was 1.39 (*p* = 0.02) and for ADV 1.80 (*p* = 0.02), both significantly increased. For obese persons, the OR for A was significantly increased to 1.72 (*p* = 0.0002) and for ADV increased to 1.72 (*p* = 0.06), just short of significance.

Table 3 shows the influence of BMI on prevalence of A or ADV, controlling for age, sex, and smoking status. After adjustments, the risk for A remained significantly elevated for obesity with an OR of 1.66 (*p* < 0.001). Although not significant, the OR for A in overweight was 1.25, (*p* = 0.12). For ADV, OR in overweight was 1.49 (*p* = 0.12) and in obesity 1.55 (*p* = 0.12), incrementally increased. The risk for A increased with age. However, there did not appear to be a relationship between age and prevalence of ADV over and above the effect of the other variables. Males had a significantly increased adjusted odds of neoplasia, with an 79% increase in OR of A (*p* < 0.001) and 173% increase in OR of ADV (*p* < 0.001) compared to females. Smoking enhanced the risk for neoplasia; this was significant for A with an OR of 1.81 (*p* < 0.001) and increased, although not significantly, for ADV with OR 1.38 (*p* = 0.26).

A family history of CRC was reported in 5.1% of all subjects, 5.0% of those without neoplasia, 5.2% with A, and 7.1% with ADV. Family history was not significantly associated with increased odds of A or ADV when considered independently or controlling for BMI, sex, age, and smoking status.

Table 4 describes the odds of neoplasia in persons with each of DM, HL, and HT, adjusted for age, gender, and smoking status, compared to increased BMI alone. Each of these elements was associated with an increased OR for neoplasia, which reached significance only for A with HT or HL. BMI provided the highest discriminatory capability of any component of MetS, although the differences were not significant (AUC = 0.630 for BMI, 0.619 for DM, 0.620 for HL, and 0.624 for HT). Moreover, since BMI, DM, HT, and HL are all associated with each other, the inclusion of all four of these risk factors, or any subset of them, did not provide significantly improved discriminatory power over BMI alone.

Neoplasia was more prevalent in males than in females.  Table 5 reports the adjusted odds by sex, related to BMI, age, and smoking status. A and ADV have been combined because of the small number of females with ADV. OR of neoplasia was significantly increased in obese females (OR = 1.56) and in overweight (OR = 1.53) and obese (OR = 1.86) males. The OR of neoplasia for overweight females was not significantly increased (OR = 1.10, *p* = 0.64). The difference in risk for neoplasia between males and females was not significant for BMI and age. For smokers, the difference between males and females was significant, with male smokers particularly vulnerable (OR = 2.17).

The influence of BMI on neoplasia formation by colonic segment is described in Table 6. The adjusted OR was increased for lesions in all three segments, but significantly so only for the left colon in overweight (OR = 1.54) and obese (OR = 1.66) and right colon in obese (OR = 1.53). There was no significant difference in comparing OR for right colon versus left, right colon versus rectum, and left colon versus rectum (numbers not shown).

Sixty-four persons (3.1%) had equivalent lesions in multiple segments, including 3 who had lesions in all 3 segments. In spite of small numbers, obese persons were significantly more likely than normal-weight persons to harbour polyps in multiple segments (OR = 2.58, *p* = 0.005).

## 4. Discussion

This prospective, community-based study examines the influence of various factors, most notably BMI, on the risk for colonic neoplasia. It was carried out in the real-world context of community practice.

The study concludes that overweight/obesity, MetS, and its constituents are associated with higher odds for A and ADV in a Canadian setting. The risk of neoplasia increases with age, male sex, and smoking, but controlling for these factors does not eliminate the strong influence of BMI. DM, HT, and HL are each associated with colonic neoplasia, but the presence of any combination of BMI, DM, HT, or HL does not significantly improve predictive power over BMI alone. In view of the suggestion elsewhere that rectal cancer is less under the influence of obesity than is the more proximal colon, we attempted to determine whether any particular segment is more strongly affected and found all segments to be at risk, with the left colon most profoundly. The rectum is less affected, but still at risk above background. Of particular interest is a strong association between obesity and adenomas at multiple sites, an observation described elsewhere [10] that underscores the enhanced potential for CRC in persons with polyps distributed through the colon. Larger studies are necessary to analyze further the anatomical distribution of obesity-related colonic neoplasia.

In terms of strengths, our study included only persons naïve to colonoscopy presenting for screening and is thereby unencumbered by screening history and the possibility of prior polypectomy, as well as the bias of symptoms, of polyp surveillance, or of occult blood positivity, circumstances that would enhance the likelihood of neoplasia. We chose not to exclude persons with a family history of CRC, who, interestingly, did not in our study have a significantly higher rate of neoplasia than those without. All data, including demographics, medical history, and colonoscopy/pathology reports, were extracted directly from patients and endoscopists' records, with no possibility of loss of fidelity inherent in questionnaires and registration data. Anthropometrics were measured directly at the time of screening.

The major limitation is sample size, which resulted in trends which failed to achieve statistical significance. In particular, the OR for neoplasia related to BMI reached significance only for A in obese persons. OR for ADV in the obese and for any neoplasia in overweight only trended toward meaningfulness. Study size also necessitated using A and ADV as cancer surrogates. A study with carcinoma as the outcome would be prohibitively large. It would require a sample size of 85,000 persons to demonstrate an OR for CRC of 1.5 for obese persons, in a population with a 0.3% prevalence of CRC and 30% prevalence of obesity.

The presence of DM, HL, and HT was defined from history provided by the subjects and their referring physicians as well as their medication list. We were not funded to carry out blood testing to refine the presence of metabolic syndrome constituents. This could have resulted in either an under- or overestimation of the true prevalence of these conditions, but we believe that the level of inaccuracy would be relatively minor. The study does not examine the longitudinal history of weight, smoking, HT, HL, or DM. Persons manifesting these phenotypes lifelong might differ in their risk for CRC from those in whom they were recently acquired. Existing longitudinal studies have included weight trend over time, but rely largely on self-reported information and thereby may be flawed [13]. The only prior study of CRC and obesity in a Canadian setting, a longitudinal case-control analysis, involved females only and used self-reported data on weight and height trends [14]. That study found no association between BMI and CRC incidence over 10.6 years of follow-up, although a subanalysis identified a significantly increased risk in premenopausal, obese subjects. Since it is generally accepted that the absolute risk and the link between CRC and BMI is stronger in males [12], this female-only study did not include the most imperiled cohort.

A number of prior publications have examined the relationship between obesity, lifestyle factors, and colorectal neoplasia. Many are longitudinal studies, most of which used self-reported information. Others are case-control, again often relying on self-reported data and inconsistent entry criteria. Others still are imaging-based, with concurrent colonoscopy and objective measures of obesity, particularly visceral, measured by abdominal CT scanning [15–18]. In spite of their heterogeneity, these studies have consistently demonstrated a risk for CRC associated with obesity, more so for males and perhaps less pronounced for rectal cancer than for more proximal parts of the colon [19, 20]. A similar association has been demonstrated between obesity measures and both advanced and nonadvanced polyps [10, 13]. Studies have considered as well the role of constituents of the MetS, specifically HT, HL, and DM, with inconsistent results, although DM would appear to be the most influential [21]. We found that all elements of MetS promote the development of colonic neoplasia.

Our study invites continuing discussion of risk stratification and screening strategies [22, 23]. Several studies have attempted to quantitate risk using risk scores incorporating various combinations of sex, lifestyle issues, family history, BMI, usage of nonsteroidal anti-inflammatory drugs, DM, and tobacco and alcohol usage as well as prior history of colorectal neoplasia [24]. These studies, whose inconsistent design and varied populations make comparison challenging, have reported ORs for colonic neoplasia of 3.84 to 5.75 between the highest echelons of risk score and the lowest. Some of them incorporated dietary habits, physical activity, and alcohol usage, all difficult to verify or quantitate. A recent US-based study, examining an average-risk population and using a scoring tool that included age, smoking, alcohol intake, height, and sex/ethnicity reported a 3.2% prevalence of ADV in its low-risk group, compared to 8.6% in its intermediate/high-risk group [25]. Interestingly, that study did not identify obesity as a risk factor and, therefore, did not include weight in its score. Another study, in a large Polish Caucasian population which had not had colonoscopy in prior 10 years, developed a score weighted largely towards age, sex, and family history, which appeared as risk factors in a test set. BMI was found to be influential, but DM did not appear to confer risk [26]. HT and HL were not considered in the database. Data was collected by questionnaire. The highest scoring subjects (maximum 9 points) had a risk of ADV of 19%, versus 1.3% and 4.5% for scores of 1 and 2, respectively. However, simply being a male over 59 years of age scored 5 out of 9, almost reaching the high-risk threshold. The authors suggested, with males twice as likely to harbour ADV, that “the man should be specifically encouraged to be screened.” While this suggestion perhaps breaches the limits of political correctness, it invites discussion of which risk factors justify intensification of screening. A smaller study, on a US population, emphasized waist circumference, sex, and tobacco usage. Other elements of MetS were not assessed. Risk of ADV ranged from 1.65% in those with score 0, to 22.3% with score >6 [27]. While a qualitative recognition that male sex, advancing age, smoking, and obesity are risk factors for CRC might suffice to generate a recommendation for preferred colonoscopy, a scoring system such as those described above might quantitatively identify at-risk individuals for whom colonoscopy could be recommended where risk stratification guides the deployment of resources.

A recent debate of the merits of risk factors as a guide to management emphasizes that any potential application would pertain only to the first screening encounter and would be most relevant in jurisdictions where primary colonoscopy is not recommended or affordable [28]. Such is the case in Ontario, where recent guidelines have recommended fecal occult blood testing as the modality of choice for average-risk asymptomatic individuals, with colonoscopy applied to those at high risk, particularly related to family history [5, 6]. However, studies such as ours indicate that other factors, including male sex, ethnicity, tobacco consumption, and obesity may be as influential as family history, yet carriers of these risk factors are not considered deserving of enhanced scrutiny.

In summary, our study demonstrates that, in a Canadian population presenting for screening colonoscopy, obesity and its sequelae are associated with an increased prevalence of benign colorectal neoplasia and, by implication, of CRC. It indicates that HT, HL, and DM each contribute to the risk for colonic neoplasia, but that BMI alone is adequate to predict risk. Thirdly, it emphasizes that obese persons are at risk for multiple colonic polyps, in which instance intensified surveillance may be appropriate, and it contributes to the overall discussion of tailoring screening strategies to individual risk. Lastly, it serves as a reminder that obesity, endemic in modern westernized society, burdens the health care system in ways beyond the familiar vascular and orthopedic consequences.

## Figures and Tables

**Table 1:** Participant characteristics; mean ± SD or *n* (%). Smoker defined as current consumption of any amount of tobacco. Family history defined as presence of at least one first-degree relative with CRC aged 60 or under.

Characteristics	All subjects	Males	Females
*N*	2,016	1,068 (53.0%)	948 (47.0%)
BMI	27.3 ± 5.1	27.7 ± 4.3	26.9 ± 5.8
Overweight	796 (38.9%)	498 (45.6%)	298 (31.2%)
Obese	526 (25.7%)	382 (25.9%)	243 (25.4%)
Hypertension (HT)	578 (28.2%)	330 (30.2%)	248 (25.9%)
Hyperlipidemia (HL)	475 (23.2%)	301 (27.6%)	174 (18.2%)
Diabetes mellitus (DM)	209 (10.2%)	134 (12.3%)	75 (7.8%)
Number of HT, HL, or DM			
1	452 (22.1%)	265 (24.3%)	187 (19.6%)
2	258 (12.6%)	160 (14.7%)	98 (10.3%)
All 3	98 (4.8%)	60 (5.5%)	38 (4.0%)
Age, years			
<50	265 (12.9%)	139 (14.5%)	126 (11.5%)
50–59	1181 (57.7%)	541 (56.6%)	640 (58.7%)
60–69	472 (23.1%)	220 (23.0%)	252 (23.1%)
≥70	129 (6.3%)	56 (5.9%)	73 (6.7%)
Smoker	286 (14.0%)	186 (17.0%)	100 (10.5%)
Family history	115 (5.6%)	57 (5.2%)	58 (6.1%)

**Table 2:** OR for A or ADV in overweight or obese persons versus normal weight, unadjusted for other participant characteristics; logistic regression.

	No neoplasia (*n* = 1,534)	Adenoma (*n* = 383)				Advanced adenoma (*n* = 99)			
	%	%	OR	95% CI	*p* value	%	OR	95% CI	*p* value
BMI									
Normal	37	28	1.00			25	1.00		
Overweight	38	40	**1.39**	**1.06**, **1.83**	**0.02**	47	**1.80**	**1.09**, **2.96**	**0.02**
Obese	24	32	**1.72**	**1.28**, **2.30**	**0.0002**	28	1.72	0.99, 2.99	0.06

**Table 3:** OR for A or ADV adjusted for BMI, age, sex, and smoking status.

	No adenoma (*n* = 1,534)	Adenoma (*n* = 383)				Advanced adenoma (*n* = 99)			
	%	%	OR	95% CI	*p* value	%	OR	95% CI	*p* value
BMI									
Normal	38	28	1.00			26	1.00		
Overweight	38	40	1.25	0.94, 1.65	0.12	46	1.49	0.90, 2.48	0.12
Obese	24	32	**1.66**	**1.23**, **2.24**	**<0.001**	28	1.55	0.89, 2.72	0.12
Age									
40–49	14	10	1.00			14	1.00		
50–59	58	57	1.14	0.77, 1.69	0.53	51	0.79	0.41, 1.52	0.48
60–69	22	25	1.30	0.84, 1.99	0.24	30	1.23	0.62, 2.47	0.55
≥70	6	8	1.69	0.98, 2.92	0.06	5	0.75	0.26, 2.20	0.60
Sex									
Female	51	35	1.00			27	1.00		
Male	49	65	**1.79**	**1.41, 2.27**	**<0.001**	73	**2.73**	**1.71, 4.35**	** <0.001**
Smoker									
No	88	79	1.00			83	1.00		
Yes	12	21	**1.81**	**1.34, 2.43**	**<0.001**	17	1.38	0.79, 2.39	0.26
Family history									
No	95	95	1.00			93	1.00		
Yes	5	5	1.09	0.65, 1.85	0.74	7	1.42	0.62, 3.26	0.41

**Table 4:** Risk of neoplasia with HT, HL, DM, or increased BMI, adjusted for age, sex, and smoking status.

	No adenoma (*n* = 1,534)	Adenoma (*n* = 383)				Advanced adenoma (*n* = 99)			
	%	%	OR	95% CI	*p* value	%	OR	95% CI	*p* value
BMI									
Normal	37	28	1.00			25	1.00		
Overweight	38	40	1.25	0.94, 1.65	0.12	47	1.49	0.90, 2.48	0.12
Obese	24	32	**1.66**	**1.23**, **2.24**	**<0.001**	28	1.55	0.89, 2.72	0.12
Diabetes									
No	91	86	1.00			87	1.00		
Yes	9	14	1.40	0.98, 1.98	0.06	13	1.27	0.68, 2.36	0.46
Hyperlipidemia									
No	78	71	1.00			74	1.00		
Yes	22	29	1.30	0.996, 1.69	0.053	26	1.08	0.67, 1.75	0.75
Hypertension									
No	73	65	1.00			68	1.00		
Yes	27	35	**1.39**	**1.08**, **1.79**	**0.01**	32	1.22	0.77, 1.93	0.39

**Table 5:** Risk of neoplasia by sex, adjusted for BMI, age, and smoking status.

	Females				Males			
	No adenoma	Any adenoma			No adenoma	Any adenoma		
	(*n* = 779)	(*n* = 160)			(*n* = 755)	(*n* = 322)		
	%	%	OR	*p* value	%	%	OR	*p* value
Sex			1.00				1.33	
BMI								
Normal	44	38	1.00		30	23	1.00	
Overweight	31	30	1.12	0.59	45	47	1.47	0.058
Obese	24	33	**1.56**	**0.04**	24	30	**1.81**	**0.0007**
Age								
<50	13	13	1.00		11	10	1.00	
50–59	58	56	0.99	0.97	60	56	1.06	0.96
60–69	23	26	1.13	0.70	22	26	1.36	0.44
≥70	6	6	1.04	0.92	6	8	1.72	0.25
Smoker								
No	90	89	1.00		86	76	1.00	
Yes	11	11	1.01	0.98	14	25	**2.16**	**<0.001**

**Table 6:** Odds ratios for any adenoma by location, related to BMI, adjusted for age, sex, and smoking status.

		Any adenoma (*n* = 418)											
	No neoplasia (*n* = 1,534)	Left colon (*n* = 182)				Right colon (*n* = 186)				Rectum (*n* = 50)			
	%	%	OR	95% CI	*p* value	%	OR	95% CI	*p* value	%	OR	95% CI	*p* value
BMI													
Normal	37	26	1.00			30	1.00			28	1.00		
Overweightht	38	45	**1.51**	**1.03, 2.21**	**0.04**	39	1.16	0.80, 1.68	0.44	46	1.42	0.72, 2.81	0.32
Obese	24	29	**1.61**	**1.06, 2.45**	**0.03**	31	**1.50**	**1.01, 2.22**	**0.04**	26	1.35	0.62, 2.91	0.45

## Abbreviations

A: Adenoma
ADV: Advanced adenoma
AUC: Area under curve
BMI: Body mass index
CRC: Colorectal cancer
DM: Diabetes mellitus
HL: Hyperlipidemia
HT: Hypertension
OR: Odds ratio
MetS: Metabolic syndrome.
